# Determinants of sanitary status among food establishments in urban setup in Adwa town, Tigray, Ethiopia: a cross-sectional study

**DOI:** 10.1186/s13104-019-4435-5

**Published:** 2019-07-12

**Authors:** Brhane Gebremariam, Berhane Asmelash, Desalegn Tetemke

**Affiliations:** 1 School of Public Health, College of Health Sciences, Aksum University, Aksum, Ethiopia; 2 Department of Environmental Health, Relief Society of Tigray (REST), Adwa, Ethiopia

**Keywords:** Sanitation, Food establishment, Ethiopia

## Abstract

**Objective:**

Food borne diseases are predominant in all parts of the world especially in urban areas and are the main source for food borne illness. The aim of this study is to assess sanitation status and its determinants among food establishments in Adwa town, North Ethiopia from March to June 2017.

**Results:**

A total of 391 (95.4%) subjects were included in this study. Around 53.3% of food establishments in the study area were in a poor sanitary status. Presence of trained managers on hygiene and sanitation (AOR = 2.6, 95% CI 1.7–4.1); inspection by regulatory personnel (AOR = 1.95, 95% CI 1.36–22.4) and being licensed (AOR = 1.2 95% CI 1.11–2.51) were associated factors which affect sanitary status sanitary of the establishments. The overall sanitary status of the establishments in the study area was found unhygienic. Managers should gain trainings on food hygiene and sanitation to follow and improve the sanitary status of the establishments.

**Electronic supplementary material:**

The online version of this article (10.1186/s13104-019-4435-5) contains supplementary material, which is available to authorized users.

## Introduction

Food borne disease contributes a great role on public health problem worldwide. A huge number of people become diseased (above 1000 million) and many of them die (37,000) every year due to contaminated foods [1].

Rising urbanization and changes in standard of living led persons to eat away from house frequently, resulting to the unrestricted establishment of food institutions which habitually have scarce sanitation environments like, unhygienic kitchen areas, poor accessibility and cleanliness of latrine facilities and lack of trainings on food handling [1]. Therefore, for the increment of the awareness of administration of the food establishments on one hand and fitness for consumers of the foods on the other hand, rating system of food sanitation status was established in different nations like New Zealand, Singapore, United Kingdom, United States, Denmark, and Canada [2].

In Ethiopia, as in other developing countries, adequate and reliable data on foodborne infectious diseases are lacking. The existing disease reporting system does not clearly and quantifiably indicate foodborne disease prevalence. Outpatient morbidity statistics (hospitals and health center’s only) of selected foodborne illnesses indicated that, the annual incidence ranged from 3.4 to 9.3% with median 5.8%. Ethiopian Demographic and Health Surveys report of 2011 and 2016 revealed that, the proportion of households with toilet facilities nationally increased from 55% in 2011 to 61% in 2016. However, the progress was significantly lower than the stipulated national target of 100% coverage [3].

Every establishment which supply food for a large group of customers has the duty to maintain the safety and wholesomeness of food otherwise it may consequence outbreaks of food and water related illness [4].

Due to the increasing consumption of food in food services establishments, such as hotels, restaurants and snack bars the community is facing different health problems like acute watery diarrhea [5]. Therefore, this study was aimed at assessing the sanitary status and its determinants among food establishments in Adwa town, Tigray, North Ethiopia.

## Main text

### Methods

#### Study area and population

The study was conducted in Adwa town, which is one of the largest towns in Tigray, located 1015 km away from Addis Ababa. It has a total of 63,759 populations. According to data obtained from the Adwa town health office, regulatory core process, there are 589 food establishments scattered in the five kebelles of Adwa town. All the food establishments, were the source population from which the study population were selected.

#### Study design and sampling method

A community based cross-sectional design was used to assess sanitary status of the food establishments and the factors which determine it. Single population proportion formula was used to determine the sample size, assuming level of sanitary status 45%, 95% confidence level, 5% margin of error and 10% of non-response rate [1]. 410 subjects were taken as a final sample size for this study. List of the establishments in the study area were used as sampling frame and the establishments were stratified by type of service they give. The aim of stratification was to make the sampling method more representative. After proportional allocation of sample size for each stratum study participants were selected using simple random sampling technique.

#### Data processing and analysis

Data were collected by trained environmental health professionals, using a structured pre-tested questionnaire and observational checklist reviewed from related articles. To assess the presence, use, cleanliness and maintenance status of sanitary materials observational checklist was used. Data were coded, checked, cleaned and corrected for errors and entered into SPSS version 20.0 and analyzed. Bivariate logistic regression were used to identify the predictor variables associated with the outcome variable at p < 0.3. The odds ratio was computed to show the strength of association of the explanatory variables and the dependent variables. Statistical significance tests were assured using odds ratio at cut-off value of 95% CI and p < 0.05.

#### Operational definition

The dependent variable of this study was sanitation status, which was calculated by taking summation of seventeen criteria’s presented in Table 2. Each criteria was given a value of 1 for the presence of sanitation facility and 0 for the absence. The sums of these available facilities were calculated and the average score of all criteria’s was used as a cut-off point to categorize establishments. Food establishments with higher than mean value were categorized under good sanitary status; whereas, those which score less than the mean or < 10.9 were considered as poor sanitary status.

#### Ethical consideration

Ethical clearance and Letter of permission was obtained from the ethical Review Board of Aksum University College of Health Sciences and Adwa town health office, respectively. Verbal and written consent was also obtained from study participants after the purpose of the study was explained.

### Results

#### Socio-demographic characteristics

Out of the 410 owners/managers of the food establishments, a total of 391 participants were interviewed with 95.4% response rate. The study assessed 63 (16.1%) hotels, 175 (44.8%) bar and restaurants, 49 (12.5%) snack house, 43 (11%) cafeterias, 13 (3.3%) butcher shops, 11 (2.8%) Juice house and 37 (9.4%) pastry shops. In the present study, among 391 establishments 354 (90.5%) were licensed. The average year of service of the establishments was 7 years, ranging from 1 year to 20 years. The median age of owners/managers was 48 years, ranging from 19 to 67 years. Only 6 (1.5%) were illiterate (Table 1).

**Table 1:** Socio-economic conditions among food establishments in urban setup, Adwa, Ethiopia, June 2017

Characteristics	Category	Frequency (n)	Percent (%)
Age	Less than 34	218	56
	34–48	154	39.6
	Above 49	19	4.0
Sex	Male	197	50.4
	Female	194	49.6
Educational status	Illiterate	6	1.5
	Grade 1–4	60	15.3
	Grade 5–8	111	28.4
	Grade 9–12	148	37.9
	Diploma and above	66	16.9
Marital status	Single	112	28.6
	Married	220	56.3
	Divorced/widowed	59	15
Establishment licensed	Yes	353	90.3
	No	38	9.7
Owner ship of establishment	Private	79	20.2
	Rented	312	79.8

#### Sanitary status of the food establishments

The sanitary status of the food establishments in the study area was observed and the status of sanitation in establishments was 53.3% 95% CI (48.4–58.5%). Three hundred seventy-five (95.6%) of the establishments had a kitchen. On the other hand, 168 (44.4%) of the establishments had a proper hand washing facility. Onsite solid waste storage containers were available in 379 (96.9%) of the establishments; one hundred thirty-nine (36.7%) of the containers were sacks, 41 (10.8%) were barrels, 135 (35.6%) were dust bins and the other 64 (16.9%) were other types of temporary use receptacles like cartons. Most of the food establishments, 385 (98.4%) had latrine facility, among them 348 (90.4%) had water washed types of toilet, and 37 (9.6%) had a dry pit latrine, of those only 38 (9.8%) were found to be with a separated unit for males and females. One hundred eighty-four (47.1%) of establishments used three compartments and the remaining 4 (1%) establishments used to wash their dishes in one compartment (Table 2).

**Table 2:** Availability of sanitation facilities of among food establishments in urban setup, Adwa, Ethiopia, June 2017

Availability of facilities	Frequency (n)	Percent (%)
Proper liquid waste disposal	288	73.7
Availability of functional hand washing facility	168	44.4
Availability of separate dressing room for food handlers	159	40.9
Properly managed latrine facility	251	64.2
Separated latrine for male and female	38	9.8
Availability of container for solid waste storage	379	96.9
Availability of piped water supply	366	93.6
Availability of three compartments for dish/glass washing	184	47.1
Availability of store room for non-perishable foods	265	67.8
Availability of separate kitchen room	375	95.6
Availability of functional refrigerator	353	90.5
Practicability of proper storage of food utensils	265	67.8
Proper drinking water storage materials	113	29.7
Food handlers wearing appropriate outer garment	126	32.2
Food handlers health examination card availability in 6 months	139	36.7
Insect or rodent infestations not found	203	51.9
Adequate ventilation of establishments	268	70.3

#### Determinants of the sanitation status of the establishments

The multiple logistic regression analysis were conducted to identify the predictor variables of sanitary status. Training on food hygiene of managers’, regulatory inspection, the presence of license, service year of the establishment were significantly associated with the sanitary status of food establishments, with p < 0.05 at 95% CI. Food hygiene trained managers own an establishment 2.6 times more likely to have good sanitary status compared to their counterparts (AOR = 2.6, at 95% CI 1.7–4.1). Food establishments which have license were 1.2 times more likely to be in good sanitary status than those which were not licensed (AOR = 1.2 at 95% CI 1.11–2.51). On the other hand, establishments which received at least one inspection visit once in a month were 1.95 times more likely to be in good sanitary status compared to those were visited once in 3 months (AOR = 1.95, at 95% CI 1.36–22.4). The study also showed establishments with less than five service year had 0.66 times less likely poor sanitary status, as compared to those with greater than eleven service year (AOR = 0.66, at 95% CI 0.36–0.92) (Table 3).

**Table 3:** Multiple logistic regression analysis of determinants of sanitary status among food establishments in urban setup, Adwa, Ethiopia, June 2017

Variables	Sanitary status		COR (95% CI)	AOR (95% CI)
	Good	Poor		
Availability of license				
Yes	353	90.3	1.97 (1.29–4.3)*	1.2 (1.11–2.51)*
No	38	9.7	1	1
Service year of establishment				
Less than 5 years	54	60	0.77 (0.47–0.98)*	0.66 (0.36–0.92)*
5–10 years	75	59	1.1 (0.68–1.75)	0.95 (0.55–1.65)
Greater than 11 years	77	66	1	1
Staff trained about food hygiene				
Yes	120	66	2.4 (1.6–3.7)**	2.6 (1.7–4.1)**
No	65	140	1	1
Regulatory inspection				
Once a months	21	27	0.28 (0.09–0.93)*	1.95 (1.36–22.4)*
Once in every 2 months	32	30	0.84 (0.43–1.75)	3.04 (0.29–11.65)
Once in every 3 months	132	105	1	1

* Significant at p < 0.05

** Significant at p < 0.001

### Discussion

In this study, absence of a license, service year of the establishment, owner/manager training on food hygiene and regulatory inspection of the food establishments were significantly associated variables (p < 0.05). This study indicated that, most of the establishments were in a state of low sanitary status (53.3%); the reasons explained might be lack of solid waste and liquid waste management practice, unimproved latrines, and inadequate drinking water accessibility and containers. This finding was lower than a study conducted in the towns of Bahir Dar which was 78.7% as well as a study conducted in Nigeria which was 69.2% [6, 7]. This variation might owed to the change in the range of development plus socioeconomic conditions of the researched site.

In this study, the proportion of food establishments operating with a formal license certificate were 90.7%. This was slightly higher than with a study done earlier at Addis Ababa which was found 70.9% and in Mekelle (86.4%) [1, 8]. The main reason could be attributed to combined effort of authorized health inspectors (regulatory bodies) and strong law and rule of the bureau of trade and investment of the town. In addition, the frequency of visit by regulatory bodies can also be explained as a main cause to the reduction of non-licensed food establishments in this study. So licensing have great contribution on sanitary status of food establishments. But, still there is lack of taking effective measures by the regulatory bodies on those who did not have license.

This study also shown that, establishments with staffs, who gain trainings on food hygiene were 2.6 times higher to keep good sanitary status of the food establishments related to their counterparts (AOR = 2.6 95% CI 1.71–4.1). Similarly, a study done by Meleko A. in Addis Ababa indicated that those who gain training on hygiene and sanitation were 1.52 more likely to have improved food establishments than those who did not gain training (AOR = 1.52 95% CI 1.05–3.0) [9]. Several studies revealed, knowledge and training of staffs on hygiene and sanitation have a direct effect on the general sanitary status of the food establishments. These had a dynamic contribution on ensuring accessibility and sanitation of sanitary materials, effective waste administration and food hygiene activities [4]. Whereas, in this study not all trained managers and food handlers keep good sanitary status of the food establishments. Kitchen, stores and latrine facilities were observed les hygienic. Food establishments which have license were 1.2 times more likely to be in good sanitary status than their counterpart (AOR = 1.2 at 95% CI 1.11–2.51). In contrast to this study, licensing had no significant association with sanitary status in study done in Bahir Dar town [7]. This difference might be explained by the level of awareness and socio-economic status of the towns. On the other hand, this finding also showed routine inspection visits by health personnel is significantly associated with sanitary status; which means, food establishments that inspected at least once in a months were 1.95 times higher than to be in good sanitary status compared to those were visited once in 3 months (AOR = 1.95, at 95% CI 1.36–22.4). Likewise, inspection of food establishments had significant association with sanitary status in a finding from Mekelle town (AOR = 2.13 at 95% CI 1.20–3.80) [8].

Majority of the food establishments in the study area require adequate sanitation facilities for food handlers. Staffs should be given an adequate number of suitable hand-washing facilities to be used by all staffs working in food handling and around premises, and the hand-washing facilities must be placed in proper and accessible position. Regular controlling mechanisms of establishments, complemented by health education and awareness creation are the proper mechanisms to advance as well as sustain hygienic status of the food establishments [6]. The finding also showed establishments with less than five service year had 0.66 times less likely to be low sanitary status, as compared to those with greater than eleven service year (AOR = 0.66, at 95% CI 0.36–0.92).

### Conclusions

In conclusion, most of the food establishments in the study area had poor sanitary status. Absence of a license, service year of the establishment, staff training on of food hygiene and regulatory inspection of the food establishment were factors associated with sanitary status. Therefore, regulatory personnel should improve and increase the frequency of their supervision for the food establishments. Similarly, the owners of the establishments should also adopt self-inspection program rather than depending on regulatory bodies alone. Simultaneously, licensing and giving training on hygiene and sanitation for managers and food handlers of the food establishments should also be improved and continuously done.

## Limitation

- Since the study was cross sectional study, it will not show cause and effect relationship and there will be observational bias.

## Additional file

**Additional file 1. ** Minimal data set used in the manuscript.

## Data Availability

The datasets used/analyzed during the study are available from the corresponding author on reasonable request (Additional file 1).

## Abbreviations

AOR: adjusted odds ratio
COR: crude odds ratio
CI: confidence interval
SPSS: Statistical Package for Social Science
